# Towards a praxis of metacognitive reflection in medical education: a framework of inquiry, adaptive action, and pattern logic

**DOI:** 10.1007/s10459-025-10469-w

**Published:** 2025-09-10

**Authors:** Jerusalem Merkebu, Stewart Mennin

**Affiliations:** 1 Department of Health Professions Education, Department of Medicine, Uniformed Services University of the Health Sciences (USUHS), 4301 Jones Bridge Road, Bethesda, MD 20814 USA; 2 Department of Cell Biology and Physiology, University of New Mexico School of Medicine, Albuquerque, New Mexico USA

**Keywords:** Metacognitive reflection, Adaptive action, Patterns, Pattern logic, Inquiry

## Abstract

Metacognitive reflection is widely recognized as essential for navigating the complexities of clinical practice and medical education. Despite extensive literature emphasizing its importance, a robust framework elucidating its practical application and theory remains a significant gap. The present paper argues that the *praxis* of metacognitive reflection—how it is enacted and acquired—is best understood and exemplified through the interconnected principles of inquiry, adaptive action, and pattern logic. These three processes offer a novel framework for both explaining the dynamics of metacognitive reflection and cultivating its development in learners and practitioners. By engaging in iterative cycles of inquiry, adapting to evolving contexts, and discerning the logic of underlying complex patterns, individuals can refine their metacognitive capacities, ultimately leading to enhanced clinical practice and learning outcomes, and a more sophisticated understanding of the complex adaptive systems within which healthcare operates. This model has the potential to significantly advance both our theoretical understanding of metacognitive reflection and its practical application in the training and professional development of health professionals.

## Introduction

Every day, learners, medical educators, and health professionals encounter novel situations when they become more aware of their own thinking, make distinctions, have ideas, and make decisions, many of which include emotional experiences. Praxis is doing with understanding; practice into theory and theory in practice (Cohen, 1996). Being aware of one’s own thinking and reflecting on it is called metacognitive reflection (MR). This experience involves sensemaking, a fundamental process that is particularly important in complex and uncertain situations. Some complex medical education examples include small-group learning (Arrow et al., 2000; Mennin, 2007, 2010) differential diagnosis (Connor et.al., Connor et al., 2022; Majumder et al., 2019); self-regulation (Cleary et al., 2016); community-based medical education (Mennin & Petroni-Mennin, 2006); formative assessment (Schuwirth & van der Vleuten, 2004); and workplace-based learning (Pangaro, 1999; Norcini & Burch, 2007). We first describe and define MR as a process and point to its relevance and benefits in health professions education and practice. We then explore understanding and developing MR as a complex adaptive event using three essential underlying mechanisms, inquiry, adaptive action, and pattern logic. Finally, we show how MR can be developed, learned, and practiced as part of health professions education.

## What is metacognitive reflection?

Metacognitive reflection is more than just thinking about one’s thinking; it is a profound and deliberate process that is essential to complex clinical decision making, research planning and analysis, small-group learning, and all stages of teaching and learning. Metacognitive reflection is a complex interplay of cognitive processes, emotional content, and reflective examination of context (Grossman, 2009; Merkebu et al., 2024; Verplanken et al., 2007). It transcends the rudimentary cognitive function of information processing, embodying instead a higher-order awareness of one’s own cognitive and learning processes. This ‘learning to learn,’ involves the critical and reflective evaluation of information. The process intensifies into metacognitive reflection when an individual’s self-assessment becomes more explicitly articulated, thoroughly elaborated, and creatively applied. At this level, it extends beyond the immediate confines of a given task to consider the broader implications and contextual significance of the intellectual endeavor (Eichbaum, 2014). Metacognitive reflection is an emergent complex cognitive process of inquiry that goes beyond mere introspection. It encompasses a critical examination of one’s cognitive processes, encompassing not only thoughts but also the emotions, awareness of context, and habits that shape and influence them (Merkebu et al., 2023, 2024).

At its core, MR is characterized by its focus on internal observation of patterns and self-regulation leading to deliberate action (Ericsson & 2004). It involves meticulous examination of knowledge, experiences, and emotions - the affective states that color our perceptions and decisions. It is bound up with and embedded in *inquiry* and the ability of learners and practitioners to question their cognitive and emotional processes, leading to a continual refinement of perspectives and enhanced understanding and decision-making abilities within specific contexts. Learning with understanding is a cornerstone of health professions education (Dewey, 1938; Glassman, 2001; Irby et al., 2010; Mennin, 2013), especially in today’s rapidly changing world where it’s not possible to learn everything.

Metacognitive reflection pushes learners beyond simple awareness of their thought processes, engaging them in cyclical reflections that bridge the gap between new information and emerging knowledge (Koufidis et al., 2020; Eoyang & Holladay, 2013). This cyclical pattern fosters deeper understanding, facilitates the identification of personal growth areas, and promotes a more meaningful connection with learning experiences. It is a deliberate and conscious process of reflection that involves critically examining one’s thoughts, experiences, and actions (Ericsson & 2004). Georghiades (2004) articulated that, “Metacognitive reflection involves the critical revisiting in the sense of noting important points of the procedures followed, acknowledging mistakes made on the way, identifying relationships and tracing connections between initial understanding and learning outcomes” (p. 371). He elaborated that this enables “informed action for rectifying the situation.” This complex process is vividly illustrated in the following clinical supervision.

Consider a faculty member in Family Medicine supervising a first-year resident’s patient presentation. As the resident speaks, the faculty member experiences more than a simple cognitive comparison to a required competency; they notice an internal pattern—a familiar feeling of unease tied to a previous, challenging case, and an awareness of the context, such as the resident’s tendency toward over-confidence. Instead of simply correcting the resident, the supervisor initiates a process of inquiry designed to make the resident’s thinking visible, asking, “Walk me through your reasoning. What were the key data points for you, and what emotions came up as you were assessing this patient?” This prompt pushes the resident beyond mere introspection, and they might articulate not just their clinical reasoning, but also the interplay of their underlying affective states and contextual pressures: “I felt rushed because the clinic was busy, and I focused on the lab results because that feels more certain to me.” Together, they see the patient, and the subsequent debrief becomes a shared exploration of the “wider implications.” They discuss how the resident seeking certainty in labs might affect patient rapport and how the supervisor’s initial unease shaped their questioning. This shared analysis of thoughts, emotions, and modus operandi exemplifies metacognitive reflection in action, a process that deliberately shifts the perspective of both learner and teacher as they engage in thinking about their own thinking and actions. Ford and Yore (2012) also highlight that these learning activities foster deeper, more adaptable approaches that, in turn, cultivate robust and enduring conceptual understanding–facilitating the seamless application of knowledge to novel situations (Ford & Yore, 2012).

## Benefits of metacognitive reflection

Metacognitive reflection promotes a deeper understanding of the learning process by encouraging individuals to question assumptions, explore alternative perspectives, and apply knowledge to new situations (Fogarty, 1994; McAlpine et al., 1999). Students can learn to more critically examine their own thinking and emotional responses throughout the continuum of their education. The more learning with understanding is done the more prepared learners are to take advantage of new learning opportunities at the frontier of their understanding. This approach fosters lifelong learning and promotes a greater understanding of the intricate connections between knowledge, experience, and the self.

For example, a small group of first-year medical students working on a problem-based case involving the sudden onset of breathlessness in a 26-year-old male as seen in the emergency room. The group has to grapple with their current understanding of the mechanics and regulation of respiration as well as the social determinants of breathlessness. Their initial discussion centers on textbook pathophysiology. The dynamic shifts, however, when one student shares her personal experience with asthma, while another connects the sensation to post-exercise dyspnea. The group engages in MR, looking at how they are thinking about the mechanism of respiration in relationship to the present patient. Instead of merely collecting these anecdotes, the group is prompted to analyze the *process* of their own thinking. A student asking, “Why did we immediately focus on pulmonary mechanics and overlook the patient’s potential anxiety?” exemplifies this shift. At the end of the session, they review their progress on their learning objectives being aware of how their thinking, limitations therein, and reflection led them to choose their specific learning objectives (fit for function) for the next group session. This self-awareness, described by Cornoldi (2014) as “intentional and aware control and strategic choice,” promotes person, task, and strategy awareness, essential skills for effective problem-solving (Jagals & Van der Walt, 2016). Furthermore, MR encourages individuals to move beyond simply accumulating knowledge, towards actively making meaning—stimulating a shift in perspective (Thomas, 2013). Metacognitive reflection encompasses an evaluation of both the processes and content of one’s thoughts (Verplanken et al., 2007) that enables learners to refine and deepen their strategies creating knowledge relevant in the present moment as in problem-based and workplace-based learning.

Not surprisingly, MR enhances performance across various work spaces and disciplines, particularly benefiting low-achieving students who gain valuable insights into their own thinking and that of their peers (White & Frederiksen, 1998, 2004; Blank, 2000). This process facilitates a deeper understanding of concepts, leading to more permanent knowledge and reduced likelihood of reverting to misconceptions (Ford & Yore, 2012). Moreover, MR has been shown to improve self-evaluation (Verplanken et al., 2007), a critical ability in small-group learning and work teams, especially in the clinical reasoning processes (Kuiper & Pesut, 2004; Hodges et al., 2024).

This introspective approach cultivates a deeper understanding of the interplay between cognition and emotion, promoting adaptability and clinical reasoning in the face of uncertainty (Graber et al., 2012; Hargreaves, 2016). It facilitates the development of robust patient-centered relationships by enhancing empathy and communication (Hodges, 2015). Physicians in training, together with their preceptors and supervisors, who engage in this practice are better equipped to recognize their own emotional responses and biases, fostering more compassionate and individualized learning experiences and patient care. Hodges et al. (2024) stated “metacognitive reflection is an invaluable part of the medical decision-making process, especially when encountering unfamiliar scenarios, and there is value in deliberately teaching this skill” (p.7). Ultimately, integrating MR into medical training and practice empowers clinicians to become more discerning diagnosticians, agile learners, and compassionate providers, thereby enhancing the quality of care (Sandars, 2009). This interpretation of MR intersects with the conceptualization of adaptive action (Eoyang & Holladay, 2013) the implications of which we develop further.

## Assumptions and processes of metacognitive reflection

How to practice MR has been much discussed in the literature (de la Croix & Veen, 2018; Merkebu, Mennin, Durning, & Samuel, 2025). Because it is a complex adaptive process, we propose it can be understood and learned through the application of inquiry, adaptive action, and pattern logic, three processes that help to explain complex adaptive systems and their relationship to medical education.

## Inquiry and metacognitive reflection

The literature on inquiry is multifaceted and ranges from curriculum change (Mann, 2011) research (Flores & Duran, 2013), faculty development (Huber, 2008), collaboration (Emihovich & Battaglia, 2000), narrative inquiry (Bleakley, 2005; Glassman, 2001), appreciative inquiry (Keefe & Pesut, 2004), clinical reasoning (Pangaro, 1999) and adaptive action (Eoyang & Holladay, 2013). Metacognitive reflection begins with inquiry and curiosity, the active pursuit of knowledge with understanding. Inquiry is so ever present in our thinking and lives that we don’t usually think about it or metacognitively reflect about it. When we do, new possibilities emerge and our choices for accessible decisions and action increase (Mennin, 2022). Inquiry is the foundation of learning and is essential for life itself. Dewey (1933) argued when operating in inquiry “we *defer* conclusion in order to *infer* more thoroughly.” How can teachers generate conditions for inquiry? Inquiry is the entree and precursor to learning in complex situations in which uncertainty predominates (Mennin, 2022). How then are we to understand such a vital process and its relationship to medical education? When we become aware of our inquiry process, we can shape and frame it so that our actions are an optimal fit for the context and environment in which we are working (*fit for function*). One approach utilizes *standing in inquiry* through four foundational principles: 1) shifting judgement into curiosity, 2) transforming conflict into shared exploration, 3) turning defensiveness into self-reflection, and 4) changing assumptions into questions (http://www.hsdinstitute.org). These principles constitute a shared responsibility for the teacher, learner, and practitioner, forming the praxis of MR that is present and active throughout health professions education.

### Turn judgement into curiosity

Dewey (1933) argued curiosity is a “positive intellectual force” that promotes alert exploration. Curiosity promotes learning and exploration of novel features and situations (Markey & Loewenstein, 2014). Learning and curiosity are about exchange and sense making, especially important in patient-practitioner interactions sensemaking involves MR (Sandars, 2009). “*Learning can be understood as a process of exchange through which ideas, experiences, knowledge, and perceptions, etc., interact so that system-wide patterns emerge, and those patterns (concepts, principles, hypotheses, new questions, etc.) influence subsequent thought and action”* (Patterson et al., 2013, p.22). Judgment forecloses conversational possibilities; curiosity creates and sustains them. A common problem in health professions education is early closure of thinking about an issue before giving due consideration to the multiple possibilities present (Johnson & Webb, 1995). In complex adaptive situations, curiosity and questions are more important than answers.

### Turn conflict into shared exploration

There will always be differences in perspective and choices for action. Metacognitive reflection leads to a greater understanding of self and other’s perspectives (Gerace et al., 2017). It is the capacity to work together to explore possibilities that is the hallmark of successful clinical and educational work. This is the basis of successful collaboration when everyone in a team is involved, when leadership is shared and exploration and inquiry span traditional boundaries and silos (Arrow & Henry, 2010). Learning is a social activity and conflict and disagreement are a normal part of it. Too little conflict can lead to “stability” or stasis. Too much can be disruptive, inhibit connections and exchange and eventually lead to chaos (Heifetz, 1994). Conflict can be best understood as difference between people, places and things. Difference holds potential energy that when released becomes kinetic and can move a system into a new paradigm that is more energetically fit as it functions (*fit for function)* (Mahmud, 2009; Eoyang & Holladay, 2013). Active learning involves shared exploration of differences necessary for effective complex group dynamics (Arrow et al., 2000). How teachers promote conditions for shared exploration, how they generate curiosity, encourage discovery and clarify understanding require both skill and creativity. Skills can be learned and continuously developed. Creativity with discipline is artful. The skillful and creative teacher generates conditions in which shared exploration of difference makes a difference to learners and to health.

### Turn defensiveness into self-reflection

In an era emphasizing continuous learning and formative assessment, defensiveness becomes a formidable barrier to growth. Having the right answer is an expectation in the health system and in medical education, and uncertainty is often viewed as a negative and insecure condition to be overcome and conquered (Tonelli, 2019). Being metacognitively reflective both as an individual and as a group or team, moves one from the withdrawal and defensiveness to the openness of learning, growth, and development (Dewey, 1938; Schwartzman, 2007). It promotes group functionality and optimizes productivity (Arrow et al., 2000). The relentless pursuit of “right answers” in medical education often casts uncertainty as a weakness, fostering a climate where defensiveness can thrive (Kim & Lee, 2018). While inherently possessing both competitive and cooperative instincts, humans may adopt protective or defensive behaviors when perceiving threats to their well-being. Defensive behavior inhibits learning, productive growth, and collaboration (Holmer, 2014; Schwartzman, 2007). We learn best when we can reflect and make sense out of novel/uncertain situations (Weick, 1995). Self-reflection is intrinsic to sense making (Sandars, 2009; Weick, 1995). This introspective approach transforms uncertainty from a threat into an opportunity, fostering openness, learning, and ultimately, professional development.

### Transform assumptions into questions

We all make assumptions about the situations we encounter. It’s natural and necessary. Few of us pause to consider and review our assumptions in the form of questions (Merkebu, Mennin, Durning, & Samuel, 2025). Questioning assumptions is a key part of differential diagnosis, interprofessional collaboration, and small-group problem-based and team-based learning. It also promotes learning and the development of new possibilities for action (Cook & Decary, 2019; Mennin, 2007). Assumptions inform our subsequent actions. The mark of a mature professional is an ability to be metacognitively reflective, to be aware of the assumptions being made and the ability to turn them into questions, into inquiry. Assumptions are based on past experiences and current expectations (Weick, 1995). Yet, every situation, every context, is unique and presents a challenge for us to adapt. An effective teacher reflects on and examines their assumptions, makes them accessible to learners and colleagues and turns them into questions that promote dialogue and action that sustain interactions promoting deeper understanding (Arrow & Henry, 2010; Smith & Glenn, 2016).

Embracing these principles promotes MR and learning that is dynamical. They guide us in making meaning, especially in clinical situations where uncertainty is dominant.

## Adaptive action and metacognitive reflection

Adaptive action and MR are synonymous. They both involve inquiry and sensemaking which are intricately intertwined with MR, establishing a synergistic relationship that facilitates effective problem-solving and learning in complex adaptive situations. Metacognitive reflection, as the foundational deliberate examination of one’s cognition, affect, and context, serves as a critical approach for each facet of the adaptive action framework. Adaptive action is an effective approach to uncertainty, when you don’t have all the information you need, when the situation is fuzzy, and decisions are required, for example, workplace-based, and problem-based and especially differential diagnosis and treatment). As elucidated by Eoyang and Holladay (2013) adaptive action presents an inquiry-based framework for navigating ambiguity and uncertainty as is done in MR. It is framed in inquiry and consists of three questions: What? So What? and Now What? In the face of uncertainty and complexity, adaptive action helps move us from observation (What?), to sensemaking (So What?), to action (Now What?). It is an iterative process such that once an action (Now What?) is taken, a new situation occurs (a new What?) where inquiry begins again with another cycle of questions.

These questions foster the development of proficient learners and practitioners who can address the uncertainty and evolving challenges of patient care and teaching. It is how preceptors learn to be teachers and how students learn to become health professionals. By thinking of MR as adaptive action, educators can empower the next generation of professionals to be more capable, adaptable, and patient-centered. A closer look at the three questions in adaptive action is useful.

## What?

The first question, What? asks what do you observe? What is going on, who is involved, where and when? What is the context of the situation? What is the data? What does the research say? What is the tension. What are the similarities and differences among the people, parts and agents involved? What holds the system together? What do you know? What do you wonder? These questions, framed as What? set the stage for So What? sensemaking and understanding, the next part of adaptive action.

## So what?

Sensemaking is the process of synthesizing information and experiences into a pattern in a cohesive narrative linking past and present (Weick, 1995; Ellaway, 2025). So What? does the data mean to me? To my team/group? To my department? To my institution? To my system? So What are the possibilities? So What is the difference that makes a difference? These are questions that are central to MR and sensemaking in clinical decision making and complex leadership. Making sense at multiple levels makes both patterns of the local context and the wholistic picture accessible. The exploration of differential diagnosis depends on MR. One can zoom in and zoom out of the situation without getting lost while finding the perspective that fits best. Moreover, the ability to make informed decisions and adapt strategies as necessary, is fortified by MR and adaptive action. The capacity for self-regulated learning and the strategic planning of educational activities is predicated upon skills in MR (Kuiper & Pesut, 2004).

## Now what?: Integrating adaptive action and medical education

Asking Now What? moves MR from sensemaking to action, to doing what is accessible now in the present moment. Now what will you do? Now what will you measure? Now what will you say? It makes adaptive action useful in complex adaptive systems and moves us from uncertainty to informed action (Eoyang & Holladay, 2013; Maitlis & Christianson, 2014). Action, however small, changes the situation and moves us to a new What? in the next cycle of adaptive action. Moving through multiple cycles of adaptive action can be understood as best practice for complex adaptive system challenges. It is an iterative process that can happen at the speed of thought or take years. It is how we move through the world, observing, perceiving, making sense, acting, and observing what happens next to begin another cycle (Eoyang & Holladay, 2013). It’s how teaching and learning go together with MR.

Clerkship directors often perceived students struggling with applying knowledge to clinical reasoning and engaging in self-regulated learning, which students rarely recognized as struggles (Irby et al., 2010). Instead, they spend their energies struggling with uncertainty in their roles and responsibilities, expectations on their clinical performance, and the logistics of clinical settings. Helping learners to perceive clearly what is going on, to make sense and meaning of the data and then to take informed action is how health professionals work.

The deliberations of a clinical curriculum committee seeking to establish grades for students in a clinical rotation require reflectively thinking about thinking. MR bolsters this alignment because individuals critically examine their thought processes, strategies, and choices in relation to the desired outcome (Georghiades, 2004; Medina et al., 2017). This conscious evaluation enables a deeper understanding of the contextual nuances, allowing for adjustments and adaptations that ensure actions are purposeful and effective. By fostering a mindful approach to problem-solving and formative assessment, MR and adaptive action facilitate the selection and execution of actions that are inherently “*fit for function*” (Eoyang & Holladay, 2013), promoting successful navigation of complex and dynamic environments

Adaptive action leverages *pattern recognition* (discerning emergent properties within a system), inquiry (active questioning to generate deeper understanding), and integration and synthesis of disparate information to arrive at a cohesive perspective (Eoyang & Holladay, 2013). Adaptive action is vital for physicians who routinely encounter unpredictable patient presentations and treatment responses (Woodruff, 2019). Pattern recognition aids diagnosis, and management, inquiry drives the thorough collection of patient information, and self-organization facilitates holistic understanding, leading to effective action. Adaptive action empowers physicians to adjust their approach based on real-time understanding and patient feedback, fostering continuous improvement in care (Woodruff, 2019). Case-based and problem-based learning cultivate inquiry and pattern recognition while encouraging active questioning during problem-based learning (Mennin, 2007). MR in collaborative discussions can facilitate the identification of the *so what* of adaptive action. Simulated scenarios can provide trainees with safe spaces to practice adaptive action. Nurturing these skills equips future physicians to manage the complexities of learning about clinical practice, promoting optimal patient care.

Effective MR and adaptation in complex systems requires the capacity to make sense of patterns, take informed action, and learn from the outcomes. Successful leaders exhibit these adaptive capacities demonstrating their ability to learn and respond effectively to changing circumstances (Mennin et al., 2020). MR foregrounds the ability of healthcare providers to accurately assess the nature and pattern of a patient’s condition, recognize when additional expertise is needed, and adapt their management strategies accordingly is critical to ensuring the best possible outcomes for patients (Merkebu et al., 2025).

## Patterns, pattern logic, and metacognitive reflection

Complexity can be understood as the interdependent interaction among multiple semi-autonomous agents such that new system-wide patterns emerge (Dooley, 1997). BecauseMR is complex, it is a self-organizing thought pattern that connects past and present with systemic patterns (Bak, 1996; Prigogine, 1996; Mennin, 2010). Conceptions of patterns (Ellaway, 2025) encompass both static, equilibrium-based structures like a crystal or a woven blanket and dynamic, far-from-equilibrium systems like the nervous system. The ability to discern and use patterns to make meaning and act in unpredictable situations is called Pattern logic. It consists of three interdependent conditions—containers (C), differences (D), and exchanges (E)—that constitute and explain the path, speed, and shape of self-organization of a pattern (Eoyang, 2001, 2003; 2004; Mennin et al., 2020).

The metaphor of a container (C) is something that holds the participants, agents, and objects involved in the pattern together long enough for them to interact and exchange differences. The *container* is the thought and reflective process of MR that is internal and yet can be open to the influence of the outside world. It is the interaction of thoughts, memories, context and system-wide influences that is important in complex patterns (Dooley, 1997; Patterson et al., 2013). The *differences* (D) between agents in the CDE model create potential energy that provides the force for change and for new patterns to emerge. In MR, it is the energy of difference between thoughts, present and past, that leads to change and action. The third component of the CDE model is exchange (E). How the differences interact and *exchange* energy, information, and matter that produce system-wide patterns. The exchanges can be strong and direct, weak, indirect, episodic, intermittent and everything in between. Patterns of MR, ideas, thoughts, concepts, and logic are shaped and influenced by these three interdependent conditions. Metacognitive reflection itself is an emergent system-wide pattern of thought that leads to action. For example, formative assessment in medical education is a pattern. It is bounded by the current context of the situation, yet open to the life history of the participants. The differences between student and teacher make a difference to both of them and their exchange, their dialogue, promotes learning and future patterns of action.

## Implications of metacognitive reflection for educators

The challenge for educators is to teach for understanding, to challenge students to think critically (MR) and in so doing to make visible and accessible how it is that they make connections, see patterns, and take informed action. MR, inquiry, adaptive action, and pattern logic form the substrate of educators’ understanding in action. Educational expertise comes from reflective practice with understanding, from perceiving and acting upon the logic of patterns of learning (Ellaway, 2025). Such awareness and practice among educators promote the creation of spaces and opportunities for learners to engage in thinking about thinking, which contributes to health and wellbeing. Teachers make decisions about what to teach, how to engage with students, and what kind of relationships to cultivate with learners at every stage of education. These decisions navigate the continuum between certainty (replication of knowledge) and uncertainty (adaptation to novel situations). In the latter case, planning teaching, writing cases that promote inquiry and sensemaking, and providing formative assessment to clinical learners requires educators to metacognitively reflect. For educators, MR is descriptive and adaptive action is the discipline of how to implement it. As educators become more aware of the role of inquiry and adaptive action, they are able to teach insightful thinking as a disciplined activity. When preceptors and supervisors are metacognitively reflective, they gain insight into their own professional development. Finally, the implications for faculty development of educators are significant in that learning to teach and learning to be skillful and aware of the process of facilitating transformative learning in the health professions is MR. By embracing this perspective early in medical school, educators can more readily design a broader array of learning interventions that empower students to acquire the habit of knowledge with understanding (Kaufman, 1985). If we are to prepare health professionals to be able to work in an increasingly uncertain future, the capacity of educators and learners to be adaptive and metacognitively aware is essential.

## Conclusion

In summary, MR is a complex interplay of cognitive processes, emotional content, and reflective examination of context. It is a deliberate process that is essential to complex clinical sensemaking and decisions, research planning and analysis, small-group learning, interprofessional work, and all stages of teaching for understanding. We propose the experience of metacognitive reflection is best exemplified and explained through inquiry, pattern logic, and adaptive action.

Inquiry is the first step in MR and in understanding complex patterns. It is the *What?* of adaptive action. Once we have identified the pattern, the thought, idea, concept, etc., of MR, we turn to making meaning, to sensemaking. This is the *So What?* of adaptive action. It is the difference that makes a difference, the D in the CDE model (Eoyang, 2001; Eoyang & Holladay, 2013). Differences contain the energy for change. The exchange (E) of energy among the differences creates new patterns of thought, new ideas, new concepts, new actions, it is the *Now What* in adaptive action. Being adaptive and using inquiry and pattern logic in the face of uncertainty are foundational skills necessary for medical education and practice in the twenty-first century and beyond (Mennin, 2016).

Medical students need to be engaged with both simple and complex pattern challenges early in medical school and continuously thereafter so that through iteration they gain familiarity and comfort with embracing ambiguity as a source of learning rather than a knot to be untied (Ellaway, 2025). Small-group problem-based, team-based, and interprofessional learning activities are a rich source of metacognitive reflection (De Backer et al., 2021) that need to be expanded and sustained to include more of the social determinants of health as well as specific objectives in the sciences. Clinical skills, during which medical knowledge and experience are encountered in applied practice, lead to inquiry through the history taking process and a curriculum with the sciences basic to medicine (Koufidis et al., 2020). Praxis, practice with understanding is a form of MR. Learning how to talk to patients, for example senior citizens, teenagers, and a child in a wheelchair requires MR. Nurturing the capacity for MR allows learners to become intricately aware that health issues rarely exist in isolation; they often involve a complex interplay of biological, psychological, social, and environmental factors. Metacognitive reflection plays a central role in addressing the uncertainties inherent in clinical practice, while simultaneously nurturing the educational development of health professional students and postgraduates. Metacognitive reflection is central to dealing with the uncertainty that is part of clinical practice and the educational. The more we use it, the more learning and practice with understanding can take place.
